# CD71 defines functionally active spermatogonial stem cells with enhanced transplantation potential in mouse testes

**DOI:** 10.1080/19768354.2026.2709175

**Published:** 2026-07-30

**Authors:** Beom-Jin Shin, Jin Seop Ahn, Jeeseung Kim, Jeongho Lee, Soontag Jung, Sang-Eun Jung, Hyo Jin Gu, Sung-Hwan Moon, Seung Hee Shin, Hyun Min Lee, Changsun Choi, Buom-Yong Ryu, Chun Jeih Ryu

**Affiliations:** a Department of Animal Science and Technology, Chung-Ang University, Anseong, Republic of Korea; b Columbia Center for Translational Immunology, Columbia University Irving Medical Center, Columbia University, New York, NY, USA; c Department of Integrative Bioscience and Biotechnology, Institute of Bioscience, Sejong University, Seoul, Republic of Korea; d Department of Food and Nutrition, Chung-Ang University, Anseong, Republic of Korea

**Keywords:** CD71, spermatogonial stem cells, transplantation, spermatogenesis, male fertility

## Abstract

Spermatogonial stem cells (SSCs) are responsible for lifelong spermatogenesis in adult males; however, their scarcity and inherent heterogeneity, coupled with the lack of robust SSC-specific surface markers, continue to impede isolation and characterization. In the present study, we found CD71, which corresponds to transferrin receptor (TfR1; encoded by *Tfrc*), a candidate marker capable of enriching for SSC populations from mouse testes. The immunohistochemistry detected co-localization of CD71 with the undifferentiated spermatogonia marker glial cell line-derived neurotrophic factor family receptor alpha 1 (GFRα1) on the seminiferous basement membrane, with approximately 81% co-localization. Consistent with this finding, the expression of *Tfrc* was up-regulated in SSC-enriched germ cell populations relative to mouse germ cell lines (GC-1 and GC-2) and somatic cell lines from testis (TM3 and TM4). Fluorescence-activated cell sorting (FACS) analysis further showed that GFRα1^+^ cells exhibited approximately 1.4-fold higher *Tfrc* mRNA expression than GFRα1^−^ cells. Similarly, CD71^+^ cells exhibited significantly higher expression of the undifferentiated spermatogonia markers *Id4*, *Lhx1*, *Gfrα1*, *Zbtb16*, and *Etv5*. Functional transplantation assays further demonstrate that CD71^high^ cells give rise to approximately 5.5-fold more colonies than freshy isolated, FACS-unsorted donor cells. Moreover, peanut agglutinin (PNA) lectin staining confirmed the normal spermatogenic differentiation within colonies derived from CD71^high^ donor cells. Our findings collectively indicate that CD71^high^ cells represent an SSC-enriched population with enhanced spermatogenic regenerative capacity and support the use of CD71 as a complementary marker for SSC enrichment and fertility restoration.

## GRAPHICAL ABSTRACT

**Figure:**
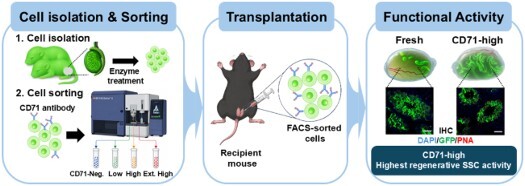

## Introduction

Spermatogonial stem cells (SSCs) are unipotent cells located on the basement membrane of the seminiferous tubules and maintain a stem cell pool that gives rise to sperm throughout male reproductive life (Ogawa et al. 2005; Kubota and Brinster 2018). SSCs can divide into themselves and spermatogonia, which are male germ cells that eventually differentiate into sperm (De Rooij and Grootegoed 1998; Oatley and Brinster 2006). Disruption in SSC maintenance or damage to the cells can deplete the stem cell pool, thereby impeding natural conception or rendering assisted reproductive technologies ineffective (Goossens et al. 2013).

Male infertility develops for many different reasons (Lee and Hwang 2024; Hwang 2025). Some causes are inherited, whereas others arise after birth because of disease, environmental exposure, or medical treatment. Among acquired causes, cancer therapy remains one of the most important because both chemotherapy and radiotherapy can severely damage the germline and impair spermatogenesis (Meistrich 2013). Improvement in cancer treatment enable more patients to survive childhood and young adolescence than in the past. As a result, preserving fertility has become an important issue following long-term care. Sperm cryopreservation before treatment is well established for post-pubertal males. However, no comparable option exists for prepubertal boys because mature sperm have not yet developed (Jensen et al. 2022). In adult patients, epididymal or testicular sperm retrieval may provide another opportunity for biological parenthood, although the outcome varies considerably according to the underlying cause of infertility (Esteves et al. 2011; Shin and Turek 2013).

As a result, SSC transplantation has therefore emerged as a promising strategy to restore spermatogenesis and fertility (Gul et al. 2020). Nevertheless, the successful clinical application of SSC transplantation depends on the efficient isolation of functional SSCs, which remains a major challenge because of the limited availability of a reliable enrichment marker (Sharma et al. 2019). At present, only a few cell-surface markers, including CD90, EpCAM, and GPR125 have been used to enrich SSCs in mice, rats, and humans, respectively (Kubota et al. 2003; Ryu et al. 2004; Nickkholgh et al. 2014; Fayomi and Orwig 2018). The identification of the additional markers capable of enriching functionally competent SSCs is therefore essential for both basic research and future clinical translation.

In finding a novel marker, cell-surface proteins involved in mechanism involving regulation of metabolic processes could be a potential candidate. Iron is required for normal cell function because it supports DNA synthesis, mitochondrial activity, and progression through the cell cycle, and redox homeostasis (Puig et al. 2017; Jiang et al. 2025). Recent evidence suggests that iron also contributes to the regulation of stem cell function. In the male germline, maintenance of iron homeostasis is necessary for normal spermatogenesis and preservation of the SSC population (Tvrda et al. 2015; Liu et al. 2022; Tsao et al. 2022). When iron homeostasis is disrupted, SSC function declines and fertility is impaired (Tsao et al. 2022; Zhang et al. 2022). Human pluripotent stem cells show a similar dependence on intracellular iron, where iron availability influences stem cell survival and cell fate through metabolic and epigenetic mechanisms (Han et al. 2019). Whether iron uptake is similarly associated with SSC function has not been established.

CD71 (transferrin receptor protein 1; TfR1, encoded by *Tfrc*) is the principal receptor responsible for transferrin-mediated cellular iron uptake. By binding iron-loaded transferrin and mediating its internalization through receptor-mediated endocytosis, CD71 supplies intracellular iron to rapidly proliferating cells (Aisen 2004; Shen et al. 2018; Wang et al. 2020). Accordingly, CD71 is essential for physiological processes requiring high proliferative activity, including erythropoiesis and neural development (Grzywa et al. 2021), and has long been used as a marker of proliferating lymphocytes and activated T cells (Schwab et al. 2020). More recently, its high expression has also been exploited for targeted drug delivery across the blood-brain barrier (Li et al. 2025). Despite these well-established roles, whether CD71 marks a functionally distinct SSC population has not been investigated.

In this study, we investigated whether CD71 could be used to enrich early spermatogonia and functional SSCs. We examined the relationship between CD71 expression and SSC regenerative capacity by spermatogonial transplantation, the gold standard functional assay for SSCs (Brinster and Avarbock 1994; Brinster and Zimmermann 1994). The results show that CD71^+^ spermatogonia are enriched for regenerative SSC activity and indicate that CD71 may be a useful complementary marker for isolating functionally competent SSCs and fertility restorations.

## Materials and methods

### Animal care

Wild type C57BL/6 (Dooyeol Biotech, Seoul, Republic of Korea) and transgenic C57BL/6-Tg(CAG-EGFP)1Osb/J mice expressing green fluorescence protein (GFP) (B6-GFP; RRID: IMSR_JAX:003291, The Jackson Laboratory, Bar Harbor, ME, USA) were used in the study. Mice were maintained under controlled conditions (23 ± 1°C and a relative humidity of 55 ± 10%, with a 12-h light/dark cycle) with free access to food and water.

### Germ cell isolation

Germ cells were isolated from 6 to 8-day-old B6-GFP pups as previously described (Lee et al. 2014). Briefly, after removal of the tunica albuginea, seminiferous tubules were washed with Dulbecco’s phosphate-buffered saline (DPBS; Cat. No. L0615, Biowest, Nuaillé, France) and dissociated into single cells using 0.25% trypsin-EDTA (Cat. No. 25200072, Gibco, Carlsbad, CA, USA) and DNase I (Cat. No. 04536282001, Roche, Basel, Switzerland) for 5 min at 37°C. Enzymatic digestion was terminated by the addition of fetal bovine serum (FBS; Cat. No. S1620, Biowest) to a final concentration of 10%, and the resulting cell suspension was passed through a 40-µm cell strainer (Cat. No. 93040, SPL Life Science, Pocheon, Republic of Korea) and centrifuged at 600 × *g* for 6 min at 4°C. The pelleted single cells were further purified by density gradient centrifugation using 30% Percoll (Cat. No. P1644, Sigma-Aldrich, St. Louis, MO, USA) at 600 × *g* for 10 min at 4°C. Subsequently, viable germ cells recovered from the Percoll gradient were washed with PBS-S buffer (PBS containing 1% FBS, 10 mM HEPES, 1 mM sodium pyruvate, 100 U/mL penicillin, and 100 μg/mL streptomycin), collected by centrifugation, and subjected to SSC enrichment using either magnetic-activated cell sorting (MACS) or fluorescence-activated cell sorting (FACS).

### Magnetic-activated cell sorting (MACS)

The single-cell suspension from germ cell isolation was resuspended in 90 μl PBS-S buffer and mixed with 10 μl Thy-1 antibody-conjugated microbeads (Cat. No. 130-049-101, Miltenyi Biotec, Auburn, CA, USA) for 15 min at 4°C in the dark, followed by MACS according to the manufacturer’s instructions, as previously described (Oatley and Brinster 2006).

Sandos inbred mouse embryo-derived thioguanine- and ouabain-resistant (STO) cells were mitotically inactivated with mitomycin C and seeded onto 0.1% gelatin-coated 24-well plates at a density of 0.1 × 10^6^ cells/well to establish feeder layers. Thy1^+^ germ cells were seeded onto feeder layers in 24-well plates at a density of 0.2 × 10⁶ cells/well. Cultures were maintained in defined mouse serum-free medium (mSFM) supplemented with 10 ng/mL recombinant human glial cell line-derived neurotrophic factor (rhGDNF; Cat. No. 212-GD, R&D Systems, Minneapolis, MN, USA), 75 ng/mL recombinant rat GDNF family receptor α1 (rrGFRα1; Cat. No. 560-GR, R&D Systems), and 1 ng/mL basic fibroblast growth factor 2 (bFGF2; Cat. No. 354060, Corning, NY, USA) (Kubota et al. 2004). Culture media were replaced every 2–3 days, and germ cells were passaged weekly onto new feeder layers. Thy-1^+^ GC-SSCs at passages 14–18 were used for reverse transcription quantitative polymerase chain reaction (RT-qPCR) analysis.

### Fluorescence-activated cell sorting (FACS)

For FACS analysis, germ cells isolated from germ cell isolation were resuspended in PBS-S buffer and incubated with either an allophycocyanin (APC)-conjugated anti-mouse CD71 antibody or an anti-GFRα1 primary antibody for 20 min at 4°C in the dark. Cells were then washed three times in PBS-S buffer, collected by centrifugation at 600 ×*g* for 6 min at 4°C, and subsequently incubated with Alexa Fluor 647-conjugated secondary antibody for 20 min at 4°C in the dark. Detailed antibody and incubation conditions are provided in Table S1.

Propidium iodide (PI; Cat. No. P4170, Sigma-Aldrich) staining and green fluorescence protein (GFP)-based gating were used to distinguish viable germ cells from dead cells during sorting on a FACSAria II flow cytometer (BD Biosciences).

CD71^+^ FACS-sorted germ cells were further divided into four populations based on their CD71 expression levels: CD71^−^, CD71^low^, CD71^high^, and CD71^extremely high^, and were used for RT-qPCR analysis or transplantation assays. GFRα1^+^ FACS-sorted germ cells were directly used for RT-qPCR.

### Culture of GC-1, GC-2, TM3, and TM4 cell lines

Mouse type B spermatogonia cell line GC-1 spg (Cat. No. CRL-2053) and spermatocyte cell line GC-2spd(ts) (Cat. No. CRL-2196) were obtained from the American Type Culture Collection (ATCC, Manassas, VA, USA). The mouse Leydig cell line TM3 (KCLB No. 21714) and Sertoli cell line TM4 (KCLB No. 21715) were acquired from the Korean Cell Line Bank (Seoul, Republic of Korea). All cell lines were maintained in Dulbecco’s modified Eagle’s medium (DMEM; LM0001-09, Biowest) supplemented with 10% FBS, 100 U/mL penicillin–streptomycin (Cat. No. 15140-122, Gibco). Cultures were maintained at 37°C in a humidified atmosphere containing 5% CO₂, with medium changes every 2 days. Cells were passaged before reaching 90% confluence. All four cell lines were used for RT-qPCR analysis.

### Reverse transcription quantitative polymerase chain reaction (RT-qPCR) analysis

RT-qPCR was conducted as previously described (Kim et al. 2025). Briefly, 1 μg of total RNA from each sample was reverse-transcribed using a reverse transcription kit (Cat. No. RP101, Biofact, Dae-Jeon, Republic of Korea) in a final reaction volume of, 20 μl. The resulting cDNA was diluted 20-fold with ddH_2_O, and 5 μl of diluted cDNA was subjected to RT-qPCR using 2× SYBR Green PCR Master Mix (Cat. No. BR122, Biofact) on a 7500 Real-Time PCR System (Applied Biosystems, Carlsbad, CA, USA). The PCR conditions were as follows: 95°C for 15 min, followed by 40 cycles of 95°C for 20 s and 60°C for 40 s, and a melt-curve analysis was subsequently performed at 95°C for 15 s, 60°C for 1 min, 95°C for 30 s, and 60°C for 15 s to confirm amplification specificity. Quantification cycle (Cq) values were normalized to 18S ribosomal RNA expression, and relative gene expression levels were calculated using the $2^{-\Delta \Delta \mathrm{CT}}$ method.

### Busulfan-induced recipient animal preparation and germ cell transplantation

To deplete endogenous germ cells from recipient testes, 5-week-old C57BL/6 mice were acclimated for 1 week. At 6 weeks of age, the mice received a single intraperitoneal injection of busulfan (45 mg/kg body weight; dissolved in a 50:50 [v/v] mixture of DMSO and ddH_2_O) 6 weeks prior to germ cell transplantation.

Germ cell transplantation was conducted as previously described (Kim et al. 2025). Briefly, CD71^+^ FACS-sorted germ cells were resuspended at a concentration of 5 × 10^6^ cells/mL in mSFM supplemented with 10% (v/v) FBS and 10 U/mL DNase I. Recipient mice were anesthetized by intraperitoneal injection of ketamine (75 mg/kg) and medetomidine (1 mg/kg), and four populations of GFP^+^ donor-derived germ cells sorted based on CD71 expression levels (CD71^−^, CD71^low^, CD71^high^, and CD71^extremely high^) were transplanted into the testes via the efferent ducts. Two months after transplantation, mice were euthanized, and testes were collected. GFP^+^ colonies (≥ 1 mm in length) were counted as previously described (Nagano et al. 1999). Colonization efficiency was calculated as the number of colonies per 10⁵ transplanted cells.

### Histology and immunohistochemistry (IHC)

Testicular tissues from age-matched 12-week-old C57BL/6 mice (healthy control) and recipient mice transplanted with CD71^+^ FACS-sorted germ cells were fixed in 4% paraformaldehyde (PFA; Cat. No. PC2031, Biosesang, Yongin-si, Republic of Korea) and embedded in paraffin. Tissue sections (4-μm thickness) were prepared for immunohistochemical analysis.

**Figure 1.**
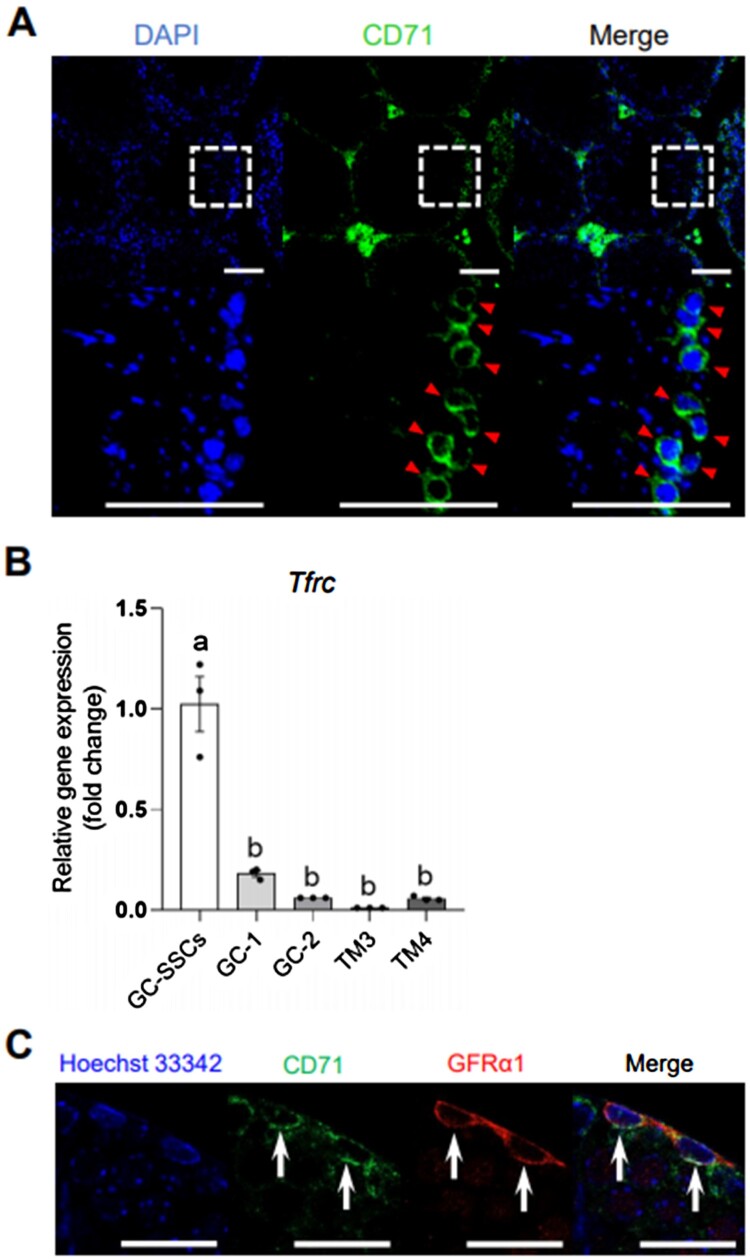
**CD71-expressing cells are localized along the basement membrane of the testis and are highly expressed in germ cells enriched for spermatogonial stem cells (GC-SSCs) (A) CD71 (green) is localized along the basement membrane of the seminiferous tubules**. The region outlined by the white dashed box is shown at higher magnification (lower panel). Red arrowheads indicate CD71^+^ cells. Nuclei are counterstained with DAPI (blue). Scale bar = 100 μm. (B) *Tfrc* expression in GC-SSCs was compared with that in GC-1 (spermatogonial-derived), GC-2 (spermatocyte-derived), TM3 (Leydig), and TM4 (Sertoli) cell lines. RT-qPCR data were normalized to 18S rRNA and are presented as mean ± SEM. Different letters (a, b) above the bars indicate statistically significant differences between groups (*p* < 0.05, n = 3). (C) CD71 (green), indicated by white arrows, co-localizes with the undifferentiated spermatogonial marker, GFRα1 (red). Nuclei are counterstained with Hoechst 33342 (blue). Scale bar = 25 μm. DAPI, 4′,6-diamidino-2-phenylindole; RT-qPCR, reverse transcription quantitative polymerase chain reaction; SEM, standard error of mean.

For antibody staining, paraffin-embedded sections mounted on glass slides were deparaffinized in xylene for 15 min and rehydrated through 100%, 90%, 70%, and 50% ethanol. Antigen retrieval was performed in 0.01 M sodium citrate buffer (pH 6.0) at 95°C for 30 min, followed by permeabilization with 0.1% Triton X-100 in PBS for 10 min at room temperature (22–25°C). Non-specific binding was blocked with 5% bovine serum albumin (BSA) in PBS for 1 h. Sections were incubated overnight at 4°C with primary antibodies diluted in PBS containing 5% BSA (IHC antibody information is also provided in Table S1). After washing, sections were incubated with appropriate Alexa Fluor secondary antibodies for 1 h at room temperature. Nuclei were counterstained with either 4′,6-diamidino-2-phenylindole (DAPI) using VectaShield Mounting Medium (LSBio, Seattle, WA, USA) or Hoechst 33342 (Cat. No. B2261, Sigma-Aldrich). Images shown in Figure 1(A) were acquired using a Nikon TE2000-U microscope equipped with NIS-Elements software (Nikon, Tokyo, Japan). All remaining images were captured using a Zeiss LSM 800 Airyscan confocal microscope with ZEN 2.6 Lite software (ZEISS, Oberkochen, Germany).

### Statistical analysis

All statistical analyses were performed using SPSS software (version 20; IBM, Armonk, NY, USA). Comparisons between two groups were analyzed using Student’s *t*-test, whereas comparison among multiple groups were performed using one-way analysis of variance (ANOVA) followed by Tukey’s honestly significant difference (HSD) *post hoc* test. Data are presented as the mean ± standard error of the mean (SEM). Differences were considered statistically significant at *p* < 0.05. All experiments were performed independently at least three times. The number of replicates for each experiment is indicated in the corresponding figure legend.

## Results

### Subcellular localization of CD71 in the testis and its expression profiles in four spermatogenic cell lines

To determine the localization of CD71 in the testis, IHC staining was performed in adult testes. CD71 was strongly localized to the basement membrane of the seminiferous tubules, where SSCs and early spermatogonia reside (Figure 1(A)). To further evaluate CD71 expression, RT-qPCR of transferrin receptor (*Tfrc*) was performed in GC-1 (spermatogonia-derived), GC-2 (spermatocyte-derived), TM3 (Leydig), TM4 (Sertoli) cell lines, using Thy-1^+^ MACS-sorted GC-SSCs as positive control. Relative to GC-SSCs (normalized to 1.02 ± 0.10), *Tfrc* expression was markedly low in GC-1 (0.18 ± 0.01), GC-2 (0.06 ± 0.00), TM3 (0.01 ± 0.00), and TM4 (0.06 ± 0.00) cells (Figure 1(B)), indicating that GC-SSCs express substantially higher levels of *Tfrc* expression than other testicular cell types.

Next, to assess whether CD71 expression is associated with established markers of undifferentiated spermatogonia, co-immunostaining for CD71 and GFRα1 was conducted following previously reported protocols (Takashima et al. 2015). CD71^+^ cells were frequently co-localized with GFRα1^+^ cells along the basement membrane (Figure 1(C)). Quantitative analysis indicated that approximately 81% of GFRα1^+^ cells co-expressed CD71, indicating substantial overlap between CD71 and GFRα1 expression.

### Expression of Tfrc in the GFRα1^+^ cell population

**Figure 2.**
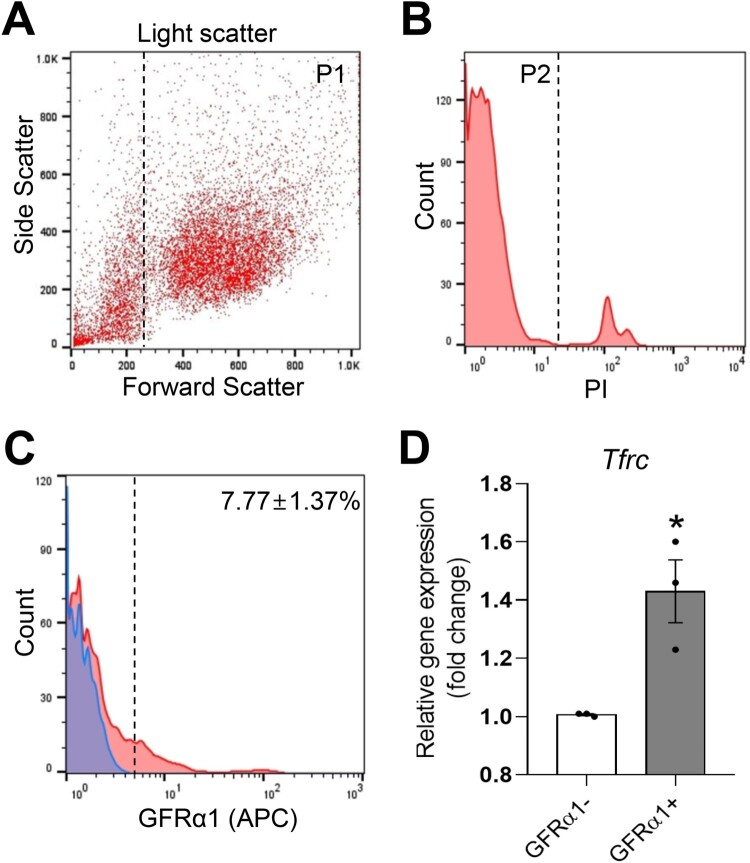
**Expression of *Tfrc* correlates with GFRα1 expression in germ cells from pup testes** (A) Pup testicular cells were first gated into the P1 fraction based on forward- and side-scatter parameters. (B) Propidium iodide^−^ (PI^−^) cells within the P1 gate were subsequently analyzed using fluorescence-activated cell sorting (FACS). (C) FACS analysis of GFRα1^+^ cells from pup testes. GFRα1^+^ cells accounted for 7.77 ± 1.37% of the total testicular cell population. (D) Comparison of *Tfrc* gene expression between GFRα1^−^- and GFRα1^+^-sorted cells. Unsorted fresh pup testis cells were used as a control. Statistical analysis was performed using Student’s t-test, and statistically significant differences between groups are indicated by an asterisk (*p* < 0.05, n = 3).

To explore the relationship between CD71 and GFRα1, testicular cells from mouse pups were first gated into the P1 fraction based on forward- and side-scatter parameters, and PI^−^ cells were selected for subsequent analysis (Figure 2(A and B)). Cells were then sorted using FACS with antibodies against GFRα1, a well-established SSC surface marker (Garbuzov et al. 2018). The GFRα1^+^ population represented 7.77 **±** 1.37% of total testicular cells (Figure 2(C)). RT-qPCR analysis indicated that *Tfrc* expression was 1.43 ± 0.08-fold higher in GFRα1^+^ than in the GFRα1^−^ cells (*p* < 0.05) (Figure 2(D)). These findings indicate a strong association between CD71 expression and GFRα1^+^ populations, supporting a potential role of CD71 in SSC identity and spermatogenic regulation.

### Expression of undifferentiated spermatogonia and germ cell-related markers in CD71-sorted cells

**Figure 3.**
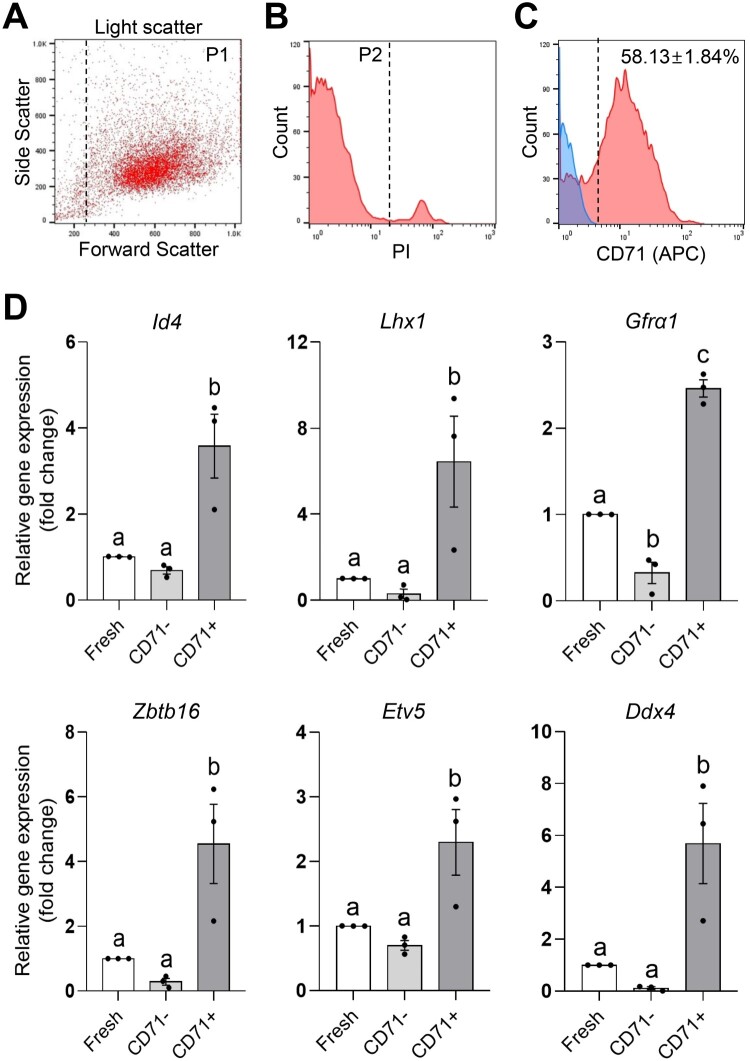
**CD71 is highly expressed in undifferentiated spermatogonia** (A) Cells isolated from pup testes were gated into the P1 fraction based on forward and side scatter characteristics. (B) Dead cells (PI^+^) were excluded, and viable P2-gated cells were subjected to subsequent flow cytometric analysis. (C) FACS analysis of CD71^+^ cells using an allophycocyanin-conjugated antibody. (D) mRNA expression levels of undifferentiated spermatogonia markers (*Id4*, *Lhx1, Gfrα1*, *Zbtb16*, and *Etv5*) and the germ cell marker (*Ddx4*) were analyzed using RT-qPCR in FACS-sorted CD71^−^ and CD71^+^ cells, with unsorted fresh cells used as a control. Data are presented as mean ± SEM. Different letters (a, b, and c) above the bars indicate statistically significant differences among groups (*p* < 0.05, n = 3). FACS, fluorescence-activated cell sorting; RT-qPCR, reverse transcription quantitative polymerase chain reaction; SEM, standard error of mean.

Pup testicular cells were analyzed using flow cytometry to isolate CD71^+^ and CD71^−^ populations. Cells were first gated based on forward and side-scatter profiles, and dead cells (PI^+^) were excluded. Viable cells were then sorted into CD71^+^ and CD71^−^ fractions for downstream analysis (Figure 3(A–C)).

RT-qPCR was performed on CD71^+^, CD71^−^, and unsorted fresh testicular cells (Figure 3(D)), using markers of undifferentiated spermatogonia, including *Id4*, *Lhx1*, *Gfrα1*, *Zbtb16*, and *Etv5* (Fayomi and Orwig 2018), as well as the pan-germ cell marker, *Ddx4* (Hickford et al. 2011).

In CD71^+^ cells, expression of germ cell-related genes was generally higher than in unsorted fresh cells. Specifically, *Id4* and *Lhx1* were increased 3.58 ± 0.47 and 6.45 ± 1.35-fold, respectively; *Gfrα1* was upregulated 2.46 ± 0.09-fold; *Zbtb16* and *Etv5* were elevated 4.55 ± 0.86 and 2.30 ± 0.33-fold, respectively. The germ cell marker, *Ddx4,* showed a 5.69 ± 0.98-fold increase. These findings indicate that CD71^+^ cells were enriched for undifferentiated spermatogonia relative to unsorted testicular cells.

In contrast, CD71^−^ cells showed lower expression of most undifferentiated spermatogonial markers compared with unsorted controls, although most changes were not statistically significant. *Gfrα1* expression was substantially reduced to 0.33 ± 0.08-fold, suggesting a selective depletion of early undifferentiated spermatogonia within the CD71^−^ fraction. Collectively, these results suggest that CD71^+^ cells were enriched in undifferentiated spermatogonia, whereas CD71^−^ cells represent a more heterogenous germ cell population with reduced undifferentiated spermatogonia content. The marked decrease in GFRα1^+^ cells within CD71^−^ fraction further supports the loss of a subset of early undifferentiated spermatogonia.

### Spermatogenic colony formation by CD71-sorted testicular cells in transplantation assays

**Figure 4.**
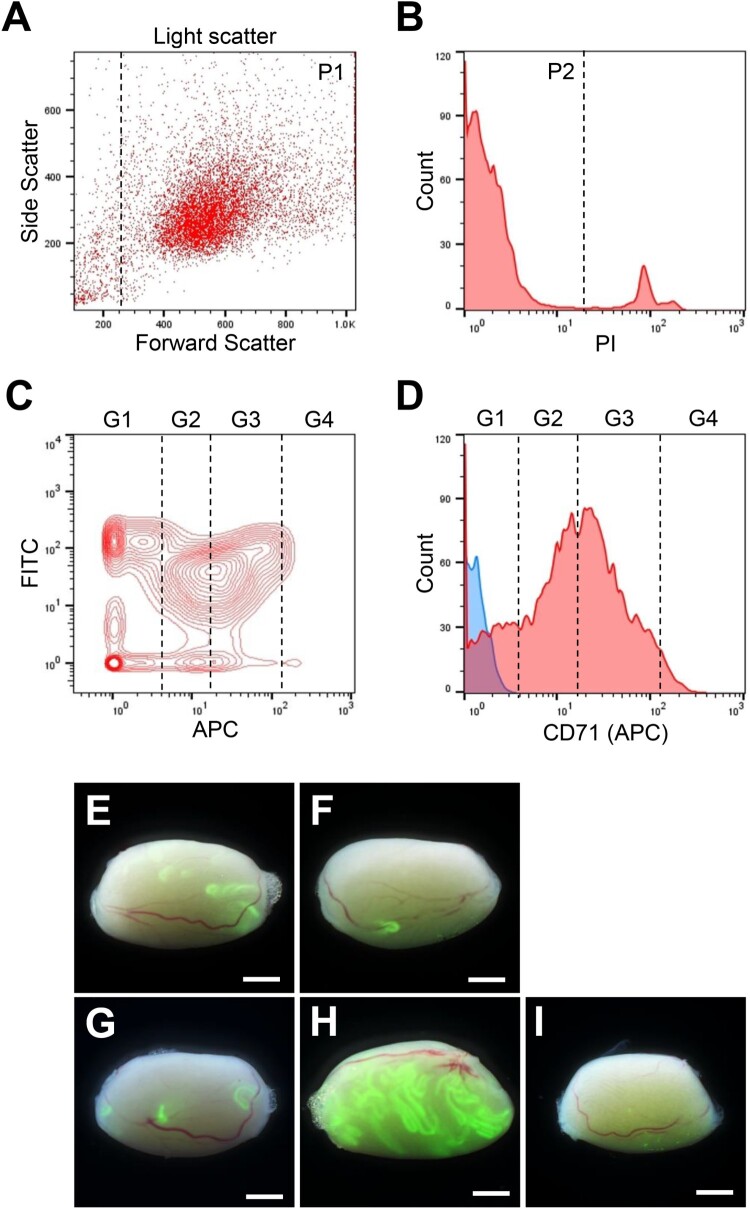
**CD71^high^ donor germ cells show enhanced spermatogenic colony formation following transplantation** (A) Flow cytometric gating of pup testis–derived cells was performed using forward- and side-scatter parameters to define the P1 fraction. (B) PI^−^ cells within the P1 gate (P2) were selected for subsequent analyses. (C) P2-gated cells were subdivided into four groups (G1–G4) based on CD71 expression levels. (D) FACS analysis of CD71 expression subdivision for transplantation. (E − I) Representative fluorescence images of recipient testes 2 months after transplantation of (E) unsorted fresh cells, and (F) CD71^−^ (G1), (G) CD71^low^ (G2), (H) CD71^high^ (G3), and (I) CD71^extremely high^ (G4) cell fractions. GFP^+^ (green) seminiferous tubules indicate colonies of spermatogenesis derived from donor stem cells. Scale bar = 2 mm. FACS, fluorescence-activated cell sorting; GFP, green fluorescent protein.

To assess the functional potential of CD71^+^ cells, pup testicular cells were gated into the P1 fraction based on forward- and side-scatter profiles, and PI^−^ viable cells were selected for analysis (Figure 4(A and B)). Cells were sorted into four populations according to their CD71 expression levels: CD71^−^ (G1), CD71^low^ (G2), CD71^high^ (G3), and CD71^extremely high^ (G4) (Figure 4(C and D)). Each fraction, with unsorted fresh cells as controls, was transplanted into busulfan-treated infertile recipient testes. Two months post-transplantation, GFP^+^ colonies were quantified (Figure 4(E–I); Table 1). Since each spermatogenic colony originated from a single transplanted SSC, the number of colonies directly reflected the presence of functional SSCs capable of self-renewal and differentiation (Oatley and Brinster 2006; Kubota and Brinster 2018).

**Table 1.** Spermatogenic potential of FACS-sorted pup testis cells by CD71 expression.

Fraction	Pup testis		
	Percentage of cells recovered (%)^a^	Colonies/10^5^ cells transplanted	Normalized no. of colonies^b^
Fresh	–	10.3 ± 2.4 (16)	
G1	40.2 ± 7.2 (3)	1.7 ± 0.8 (13)	0.7
G2	24.6 ± 1.7 (3)	9.4 ± 2.4 (18)	2.3
G3	33.8 ± 6.5 (3)	56.5 ± 9.9 (18)	19.1
G4	0.6 ± 0.0 (3)	5.6 ± 3.2 (12)	0
Total fractionated	99.1 ± 0.4 (3)		22.1

Values are presented as mean ± SEM. The number of observations is indicated in parentheses.

^a^ Cell distribution is shown as a percentage of P1 and PI^-^ (Figure 4(A and B)) in pup testis cells fractionated by FACS.

^b^ Nomalized colony number was calculated by multiplying column 2 (% of cells recovered) × column 3 (colonies per 10^5^ cells transplanted).

Of the sorted fractions, the G3 (CD71^high^) population exhibited the highest colony-forming capacity, indicating strong SSC enrichment. In contrast, G1, G2, and G4 fractions produced markedly fewer colonies, suggesting lower SSC content (Figure 4(H); Table 1). Unsorted fresh cells yielded 10.3 ± 2.4 colonies per 10⁵ transplanted cells, serving as the baseline SSC activity in pup testes.

The G1 fraction represented the largest proportion of recovered cells (40.2 ± 7.2%) but generated only 1.7 ± 0.8 colonies per 10⁵ cells (normalized 0.7), indicating limited SSC potential despite its abundance. The G2 fraction (24.6 ± 1.7%) produced 9.4 ± 2.4 colonies per 10⁵ cells (normalized 2.3), showing moderate SSC enrichment. In contrast, G3 fraction (33.8 ± 6.5%) generated 56.5 ± 9.9 colonies per 10⁵ cells, corresponding to a normalized value of 19.1, indicating a substantial enrichment of SSCs. The G4 fraction, although small (0.6 ± 0.0%), yielded 5.6 ± 3.2 colonies per 10⁵ cells (normalized 0), suggesting that excessively high CD71 expression does not enhance SSC enrichment.

Overall, the sorted fractions accounted for 99.1 ± 0.4% of total recovered pup testicular cells, yielding a cumulative normalized colony number of 22.1. The CD71^high^ cells (G3) harbored the majority of SSCs capable of self-renewal and differentiation, validating CD71 expression as a reliable criterion for SSC enrichment in transplantation assays.

### Reconstitution of spermatogenesis by CD71^high^-sorted testicular cells

**Figure 5.**
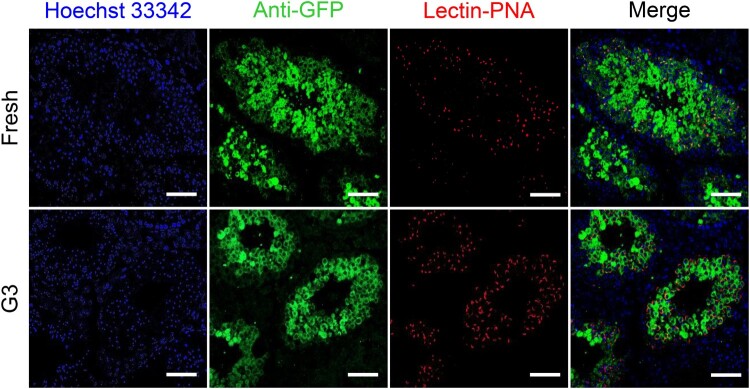
**CD71^High^ (G3) cells reconstitute donor-derived spermatogenesis in transplanted testes**. Representative fluorescence immunohistochemistry images show germ cell colonies derived from CD71^high^ (G3) cells, similar to control colonies formed by unsorted fresh cells within recipient seminiferous tubules. Spermatogenic differentiation of both unsorted fresh cells and CD71^high^ (G3) GC-SSCs was confirmed using lectin–peanut agglutinin (PNA) staining. Functional spermatogenic colonies derived from the transplanted cells were observed throughout the seminiferous tubules. Nuclei were counterstained with Hoechst 33342 (blue), and donor-derived germ cells were visualized using GFP expression. Scale bar = 50 μm. GFP, green fluorescent protein.

CD71^high^ FACS-sorted donor germ cells (G3) were evaluated for their spermatogenic potential following transplantation into busulfan-treated recipient testes. To confirm donor-derived spermatogenesis, immunofluorescence analyses were conducted two months post-transplantation using lectin–peanut agglutinin (PNA) staining, Hoechst 33342 counterstaining, and anti-GFP immunostaining (Figure 5). GFP^+^ cells were detected throughout the seminiferous epithelium, confirming successful donor-derived engraftment.

Both freshly isolated donor germ cells (Fresh) and CD71^high^ (G3) populations exhibited robust engraftment and normal spermatogenic differentiation within recipient seminiferous tubules. Lectin–PNA staining indicated strong labeling of acrosome-positive spermatids and spermatozoa in both Fresh and G3 groups, indicating efficient progression through spermiogenesis. Collectively, these results demonstrate that Fresh and CD71^high^ (G3) germ cell populations exhibited comparable spermatogenic capacity, including self-renewal and complete differentiation within the seminiferous epithelium.

## Discussion

The male fertility foundation is SSCs due to their capacity for self-renewal and differentiation, which are the precursor cells for male spermatozoa. However, SSC populations are heterogeneous, and a single definitive marker is yet to be discovered (Kubota et al. 2003; Yoshida et al. 2007; Hermann et al. 2011; Chan et al. 2014). CD71 was evaluated (although not lineage-specific to germ cells) as a valuable surface marker for the identification and enrichment of SSCs in the mouse testis. CD71-mediated iron uptake is important for spermatogenesis and meiotic progression (Gao et al. 2021). Thus, the maintenance of iron homeostasis is essential for male reproduction, while both deficiency and excess have been shown to impair testicular function (Tsao et al. 2022; Harrer et al. 2025). Iron deficiency causes a reduction in testosterone, impairs sperm production, and increases apoptosis of germ cells (Tsao et al. 2022). In contrast, iron overload causes oxidative stress and ferroptosis, resulting in infertility (Yuan et al. 2023; Harrer et al. 2025).

We observed that CD71^+^ cells are localized within the basal compartment of the seminiferous tubules, where undifferentiated spermatogonia typically reside. Similarly, cells that express GFRα1, a key receptor in the GDNF/RET that regulates SSC self-renewal and differentiation, are also predominantly found in the basal membrane. Co-immunostaining of seminiferous tubule specimens showed that CD71 was markedly colocalized with GFRα1. Approximately 81% of spermatogonia co-expressed CD71. Consistent with these findings, *Tfrc* was significantly enriched in germ cell-enriched SSC populations compared to spermatogonia cell lines (GC-1, GC-2), Leydig cells (TM3), and Sertoli cells (TM4). Likewise, GFRα1^+^ cells showed elevated *Tfrc* expression than GFRα1^−^ cells. In addition, CD71^+^ cells also showed significantly increased expression of undifferentiated spermatogonia markers (*Id4*, *Lhx1*, *Gfrα1*, *Zbtb16*, and *Etv5*) as well as the germ cell marker *Ddx4*, compared to CD71^−^ cells and freshly isolated unsorted controls. The marked decrease in GFRα1 expression in CD71^−^ population compared to the fresh controls further supports the notion of a positive correlation between *Tfrc* and *Gfrα1* expression. Both CD71 and GFRα1 are cell-surface receptors, and the enrichment of SSC self-renewal and maintenance genes, including *Lhx1* and *Zbtb16* (Oatley et al. 2006; Grasso et al. 2012; Garbuzov et al. 2018), in CD71^+^ and GFRα1^+^ populations support the use of CD71-based cell isolation as a promising approach for SSC identification and enrichment.

To further validate the results, SSC transplantation assay—the gold standard for evaluating functional SSCs was conducted. It facilitates the *in vivo* assessment of their regenerative potential (Brinster and Avarbock 1994; Brinster and Zimmermann 1994). Given that only SSCs can colonize busulfan-treated infertile recipient testes to restore spermatogenesis, identifying and enriching undifferentiated subpopulations is a prerequisite for effective transplantation (Kubota et al. 2003; Ryu et al. 2004). GFRα1^−^ cells exhibit lower transplantation efficiency compared to GFRα1^+^ cells (Buageaw et al. 2005; Garbuzov et al. 2018).

The transplantation assay gave a different picture from that obtained by phenotypic analysis alone. Few colonies developed from CD71^−^ cells, whereas the CD71^high^ (G3) fraction consistently produced the largest number of colonies. We considered both the number and the quality of regenerated colonies. Colony number reflects the frequency of functional SSCs, whereas colony quality indicates whether those SSCs retain the capacity to regenerate complete spermatogenesis after transplantation. Colonies derived from CD71^high^ cells contained GFP^+^ donor germ cells throughout the seminiferous tubules and progressed to produce PNA^+^ spermatids and spermatozoa. This indicated that the transplanted cells retained the ability to complete spermatogenesis after colonization of the recipient testis. We did not expect CD71 expression by itself to define SSC function. Nevertheless, the transplantation assays suggest that cells expressing high levels of CD71 are more likely to contain functional SSCs than the remaining spermatogonial population.

The transplantation results suggest that SSC regenerative activity does not increase in parallel with CD71 expression. Although the CD71^high^ fraction showed the greatest regenerative activity, further increases in CD71 expression were not accompanied by additional colony formation. We did not anticipate this result because a simple relationship between CD71 expression and SSC activity would have predicted the opposite. One possible explanation is that increasing CD71 expression reflects changes in the biological state of spermatogonia rather than progressive enrichment of SSCs. Cells expressing extremely high levels of CD71 may already be entering early differentiation or undergoing changes in intracellular iron metabolism. The present experiments were not designed to distinguish between these possibilities. Nevertheless, the transplantation data indicate that differences in CD71 expression are associated with functional heterogeneity within the undifferentiated spermatogonial population.

Our initial interest was simply to determine whether CD71 could improve enrichment of functional SSCs. The transplantation results suggest that CD71 expression is associated with SSC regenerative activity rather than with an undifferentiated phenotype alone. This distinction is important because the SSC function cannot be predicted reliably from marker expression by itself. We therefore consider CD71 to be a useful addition to, rather than a replacement for, existing SSC markers. Used in combination with established marker panels, CD71 may improve recovery of functionally competent SSCs. Because reliable enrichment of functional SSCs remains one of the major obstacles in SSC research, incorporating CD71 into current isolation strategies may not only increase recovery of regenerative SSCs but also facilitate studies of functional heterogeneity within the undifferentiated spermatogonial population.

## Supplementary Material

Supplemental Tables.docx

## Data Availability

All data presented in this study are available on request from the corresponding author.
