# Supplementary material for: CD71 defines functionally active spermatogonial stem cells with enhanced transplantation potential in mouse testes
Source: Anim Cells Syst (Seoul). 2026 Jul 30;30(1):448–61. doi: 10.1080/19768354.2026.2709175 (PMC13425539; doi:10.1080/19768354.2026.2709175)
Supplement: Supplemental Tables.docx [file TACS_A_2709175_SM7898.docx]

**Supplemental Tables**

**Table S1. Antibodies used for fluorescence-activated cell sorting (FACS), magnetic-activated cell sorting (MACS), and immunohistochemistry (IHC)**

| **Purpose** | **Antigen** | **Product name** | **Cat. #** | **RRID** | **Company** |
| --- | --- | --- | --- | --- | --- |
| FACS | CD71 | APC-conjugated anti-mouse CD71 | 113820 | AB_2728134 | BioLegend |
| FACS | GFRα1 | Anti-GDNF receptor alpha 1 | Ab8026 | AB_306208 | Abcam |
| MACS | Thy1.2 | CD90.2 microbeads | 130-049-101 | AB_3073748 | Miltenyi Biotec |
| IHC | CD71 | Anti-Transferrin receptor | Ab84036 | AB_10673794 | Abcam |
| IHC | GFRα1 | Rat GFR alpha-1/GDNF R alpha-1 | AF560 | AB_2110307 | R&D Systems |
| IHC | GFP | Anti-GFP | Ab290 | AB_303395 | Abcam |
| IHC | Lectin PNA | Lectin PNA From *Arachis hypogaea* (peanut), Alexa Flour 647 Conjugate | L32460 | AB_3099671 | Invitrogen |
| IHC | Secondary antibody | Anti-rabbit IgG Alexa Fluor 488 | A21206 | AB_2535792 | Invitrogen |
| IHC |  | Donkey anti-Goat IgG Alexa Fluor 568 | A11057 | AB_2534104 | Invitrogen |
| FACS |  | Anti-rabbit IgG Alexa Flour 647 | A21245 | AB_141775 | Invitrogen |

**Table S2. Primers used for RT-qPCR**

| **Gene** | **Forward primer 5’→3’** | **Reverse primer 5’→3’** |
| --- | --- | --- |
| ***Tfrc*** | TCATACACCCGGTTTAGCCTT | GCCTTCATGTTATTGTCGGCAT |
| ***Lhx1*** | CCCATCCTGGACCGTTTCC | CGCTTGGAGAGATGCCCTG |
| ***Id4*** | CAGTGCGATATGAACGACTGCT | CCAGGATGTAGTCGATAACGTG |
| ***Gfrɑ1*** | GCACCAAGTACCGCAACT | GCGGCAGTTGTAGAGAGACTTC |
| ***Zbtb16*** | CTGCGGAAAACGGTTCCTG | GTGCCAGTATGGGTCTGTCT |
| ***Etv5*** | TCAGTCTGATAACTTGGTGCTTC | GGCTTCCTATCGTAGGCACAA |
| ***Ddx4*** | GCTTCATCAGATATTGGCGAGT | GCTTGGAAAACCCTCTGCTT |
| ***18s*** | ACCTGGTTGATCCTGCCAGGT | TAGGAGAGGAGCGACCAA |
